# Control of HPV-associated tumors by innovative therapeutic HPV DNA vaccine in the absence of CD4+ T cells

**DOI:** 10.1186/2045-3701-4-11

**Published:** 2014-03-04

**Authors:** Shiwen Peng, Liwen Song, Jayne Knoff, Joshua W Wang, Yung-Nien Chang, Drew Hannaman, T-C Wu, Ronald D Alvarez, Richard BS Roden, Chien-Fu Hung

**Affiliations:** 1 Department of Pathology, Johns Hopkins Medical Institutions, Baltimore, MD, USA; 2 Department of Obstetrics and Gynecology, Johns Hopkins Medical Institutions, Baltimore, MD, USA; 3 Department of Molecular Microbiology and Immunology, Johns Hopkins Medical Institutions, Baltimore, MD, USA; 4 Department of Oncology, Johns Hopkins Medical Institutions, Baltimore, MD, USA; 5 Pharmacy School of Fudan University, Shanghai, China; 6 Department of Pharmacology and Toxicology, Shanghai Institute of Planned Parenthood Research, Shanghai, China; 7 Department of Obstetrics and Gynecology, Shanghai Tenth People’s Hospital of Tongji University, Shanghai, China; 8 Papivax LLC, Severna Park, MD, USA; 9 Ichor Medical Systems, Inc., San Diego, California, USA; 10 Department of Obstetrics and Gynecology, University of Alabama at Birmingham, Birmingham, AL, USA; 11 Departments of Pathology and Oncology, The Johns Hopkins University School of Medicine, CRB II Room 307, 1550 Orleans Street, Baltimore, Maryland 21231, USA

**Keywords:** DNA vaccine, Human papillomavirus, Immunosuppression, CD4 depletion, Calreticulin, Immunotherapy

## Abstract

Human papillomavirus (HPV) infections are particularly problematic for HIV + and solid organ transplant patients with compromised CD4+ T cell-dependent immunity as they produce more severe and progressive disease compared to healthy individuals. There are no specific treatments for chronic HPV infection, resulting in an urgent unmet need for a modality that is safe and effective for both immunocompromised and otherwise normal patients with recalcitrant disease. DNA vaccination is attractive because it avoids the risks of administration of live vectors to immunocompromised patients, and can induce potent HPV-specific cytotoxic T cell responses. We have developed a DNA vaccine (pNGVL4a-hCRTE6E7L2) encoding calreticulin (CRT) fused to E6, E7 and L2 proteins of HPV-16, the genotype associated with approximately 90% vaginal, vulvar, anal, penile and oropharyngeal HPV-associated cancers and the majority of cervical cancers. Administration of the DNA vaccine by intramuscular (IM) injection followed by electroporation induced significantly greater HPV-specific immune responses compared to IM injection alone or mixed with alum. Furthermore, pNGVL4a-hCRTE6E7L2 DNA vaccination via electroporation of mice carrying an intravaginal HPV-16 E6/E7-expressing syngeneic tumor demonstrated more potent therapeutic effects than IM vaccination alone. Of note, administration of the DNA vaccine by IM injection followed by electroporation elicited potent E6 and E7-specific CD8+ T cell responses and antitumor effects despite CD4+ T cell-depletion, although no antibody response was detected. While CD4+ T cell-depletion did reduce the E6 and E7-specific CD8+ T cell response, it remained sufficient to prevent subcutaneous tumor growth and to eliminate circulating tumor cells in a model of metastatic HPV-16+ cancer. Thus, the antibody response was CD4-dependent, whereas CD4+ T cell help enhanced the E6/E7-specific CD8+ T cell immunity, but was not required. Taken together, our data suggest that pNGVL4a-hCRTE6E7L2 DNA vaccination via electroporation warrants testing in otherwise healthy patients and those with compromised CD4+ T cell immunity to treat HPV-16-associated anogenital disease and cancer.

## Introduction

Of the over one hundred known Human papillomaviruses (HPVs), a dozen high risk types are associated with cancer. These oncogenic types infect the genital mucosa and are sexually transmitted. The oncogenic HPVs are the primary etiologic agents of cervical cancer and are also known to cause a subset of head and neck, vaginal, vulvar, anal and penile cancers [1-3]. HPV-16 is the most problematic type as it causes at least half of all cervical cancers and the great majority (approximately 90%) of the HPV-associated cancers at the other anogenital sites and oral cavity. HPV-associated diseases are a significant problem for HIV+ and otherwise immunocompromised patients who are less able to clear their HPV infections than healthy individuals. HPV-associated cancer incidence is significantly elevated at multiple sites in HIV + patients, notably cervical cancer, which has been designated an HIV-associated malignancy [4], and anal cancer that is predominantly driven by HPV-16 [5]. In addition, HIV + patients more frequently acquire multi-type infections; many of which are infrequently seen in healthy individuals, and consequently are not targeted by the current HPV vaccines [6]. Furthermore, benign HPV disease is also more severe and intractable in HIV + and solid organ transplant patients [4]. There are no specific treatments for chronic HPV infection, and warts are typically treated with limited success by surgery, ablation via cryotherapy or non-specific immune modulators. Therefore, there remains an urgent need for a therapy to effectively treat chronic oncogenic HPV infection, particularly HPV-16, and associated diseases using an approach that is safe and effective even for immunocompromised patients [7].

One potential strategy to treat HPV infection and disease in immunocompromised patients is DNA vaccination. DNA vaccines elicit cell-mediated and/or humoral immune responses and are considered safe even for immunocompromised individuals because they do not contain live pathogen [8]. Although intramuscular (IM) DNA injection has a low efficiency of host cell transduction and encoded antigen expression on its own, delivery is greatly enhanced with immediate electroporation at the injection site [9]. We have previously generated various therapeutic HPV DNA vaccines encoding HPV antigens and including various enhancement strategies such as targeting the antigen to the endosomal/lysosomal compartment [10], as well as linkage to HSP70 [11], to the extracellular domain of Fms-like tyrosine kinase 3-ligand [12] and to the translocation domain of a bacterial toxin [13]. In particular, we have found that linkage to the heat shock protein calreticulin (CRT) in DNA vaccines potently enhances the immune response to heterologous antigens [14-16]. Indeed, one of these DNA vaccines encoding CRT linked to HPV-16 E7 antigen (CRT/E7(detox)) has advanced to clinical trials. The CRT/E7(detox) DNA vaccine is currently being tested in Phase I clinical trials in patients with HPV-16+ head and neck cancer (NCT01493154) and in a trial in HPV-16+ grade 2/3 cervical intraepithelial neoplasia (NCT00988559).

Although the clinical grade CRT/E7 (detox) DNA vaccine has been used in the clinic, it could be potentially improved by the fusion of additional HPV antigens. It has been shown that T cell-mediated immune responses against HPV E6 antigen are associated with a favorable clinical outcome, and may be immunodominant in some patients [17,18]. Furthermore, humoral immune responses against the L2 capsid protein have been shown to provide broad protection against infection by a wide spectrum of HPV subtypes in animal models [19,20]. Therefore, we developed a preventive and therapeutic HPV DNA vaccine encoding CRT linked to HPV-16 E6, E7 and L2 proteins (pNGVL4a-hCRTE6E7L2) [21]. The cross-protective effects of L2-specific neutralizing antibody have the potential to prevent the unusual HPV types and multi-type HPV infections seen in the HIV + population. Indeed, we previously found that vaccination with pNGVL4a-hCRTE6E7L2 DNA vaccine was capable of generating HPV neutralizing antibodies as well as control of HPV-16 E6/E7-expressing tumors in a preclinical model [21]. In contrast to patients, E6 is poorly immunogenic in the C57/BL6 mouse, but immunization with a DNA vaccine expressing E6 fused to CRT elicits therapeutic responses [15]. Taken together, the pNGVL4a-hCRTE6E7L2 DNA vaccine has promise for the treatment of HPV16-associated lesions as well as to generate a pan-HPV protective effect against new or re-infection. However, it is unclear whether the vaccine will be active in patients with compromised CD4+ T cell-dependent immunity who so urgently need such a treatment. In the current study, we test the immunogenicity and therapeutic effects of the pNGVL4a-hCRTE6E7L2 DNA vaccine against an E6/E7-expressing murine model of anogenital HPV-16+ cancer in both immunocompetent and immunosuppressed settings.

## Results

### *In vivo* electroporation enhances cell-mediated and humoral HPV antigen-specific immune responses to intramuscular vaccination with CRTE6E7L2 DNA

**Figure 1:**
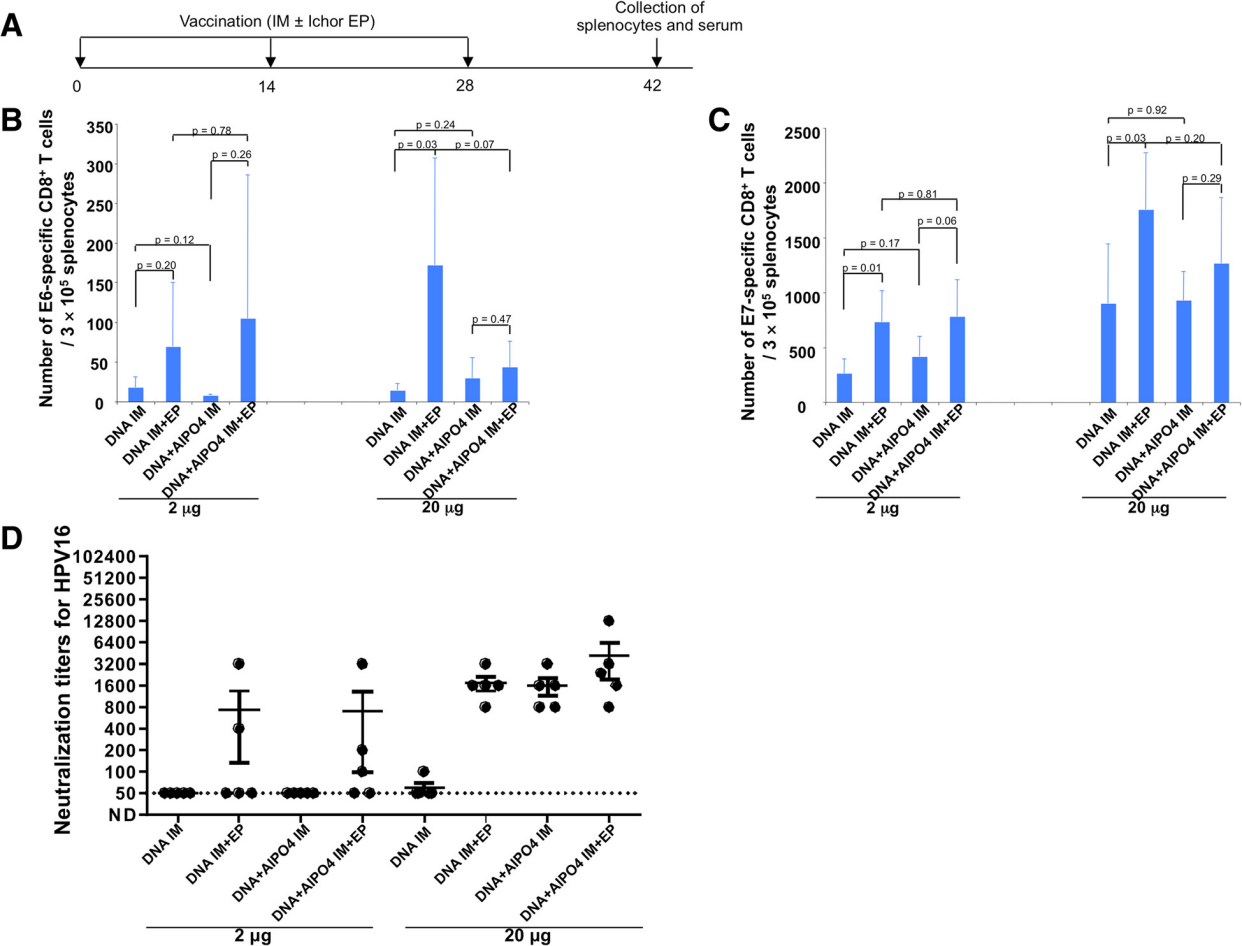
**Comparison of immunogenicity of CRT/E6E7L2 DNA vaccine administered by various methods. (A)** Schematic illustration of the experiment. Briefly, 5-8 week old female C57BL/6 mice (5 mice/group) were vaccinated with either 2 μg/mouse or 20 μg/mouse of CRT/E6E7L2 DNA, or CRT/E6E7L2 DNA formulated with 45 μg of aluminum phosphate by intramuscular injection, or followed by electroporation. The mice were boosted with the same regimen twice with a 2-week interval. Two weeks after last vaccination, serum and splenocytes were collected. Summary of HPV16 E6- **(B)** or E7- **(C)** specific CD8^+^ T cell responses analyzed by IFN-³ intracellular staining. Splenocytes were stimulated with 1 μg/ml of HPV16 E6aa48-57 or HPV16 E7aa49-57 peptide at the presence of GolgiPlug (1 μl/ml) overnight at 37°C. The cells were then stained with anti-mouse CD8 followed by intracellular IFN-³. The data were acquired with FACSCalibur and analyzed with CellQuest. **(D)** Summary of the HPV16 L2-specific neutralizing antibody responses.

In the current study, we first sought to determine the ideal route of administration of the CRTE6E7L2 DNA vaccine. C57BL/6 mice were vaccinated three times at two-week intervals with CRTE6E7L2 DNA at doses of 2 μg or 20 μg and either with or without alum (Figure 1A). The vaccines were administered intramuscularly with or without electroporation. Two weeks after the last vaccination, splenocytes and serum were collected from treated mice and analyzed by CD8+ T cell intracellular cytokine expression and HPV-16 fcPsV neutralization assays, respectively. As shown in Figure 1B and C, in general, IM administration of the CRTE6E7L2 DNA vaccine with electroporation was significantly better for generating HPV antigen-specific CD8+ T cells compared to IM administration of the DNA without electroporation. This was true for both E6 and E7, and was generally consistent between the low and high dose DNA vaccine groups. Furthermore, we observed that alum did not further enhance the generation of antigen-specific T cells elicited by IM injection of CRTE6E7L2 DNA vaccine with electroporation (Figure 1B and C). In addition, as shown in Figure 1D, at a dose of 20 μg, vaccination with CRTE6E7L2 DNA with either alum or electroporation generates similar levels of HPV-specific antibodies, and CRTE6E7L2 DNA vaccine administration with the combination of alum and electroporation only generates a minimal increase in antibody levels compared to vaccination with either DNA with alum or DNA with electroporation. Overall, these data suggest that DNA vaccination followed by electroporation generates a superior HPV-specific immune response compared to IM injection alone or with alum.

### CRTE6E7L2 DNA vaccine administered intramuscularly followed by electroporation generates potent antitumor effects

**Figure 2:**
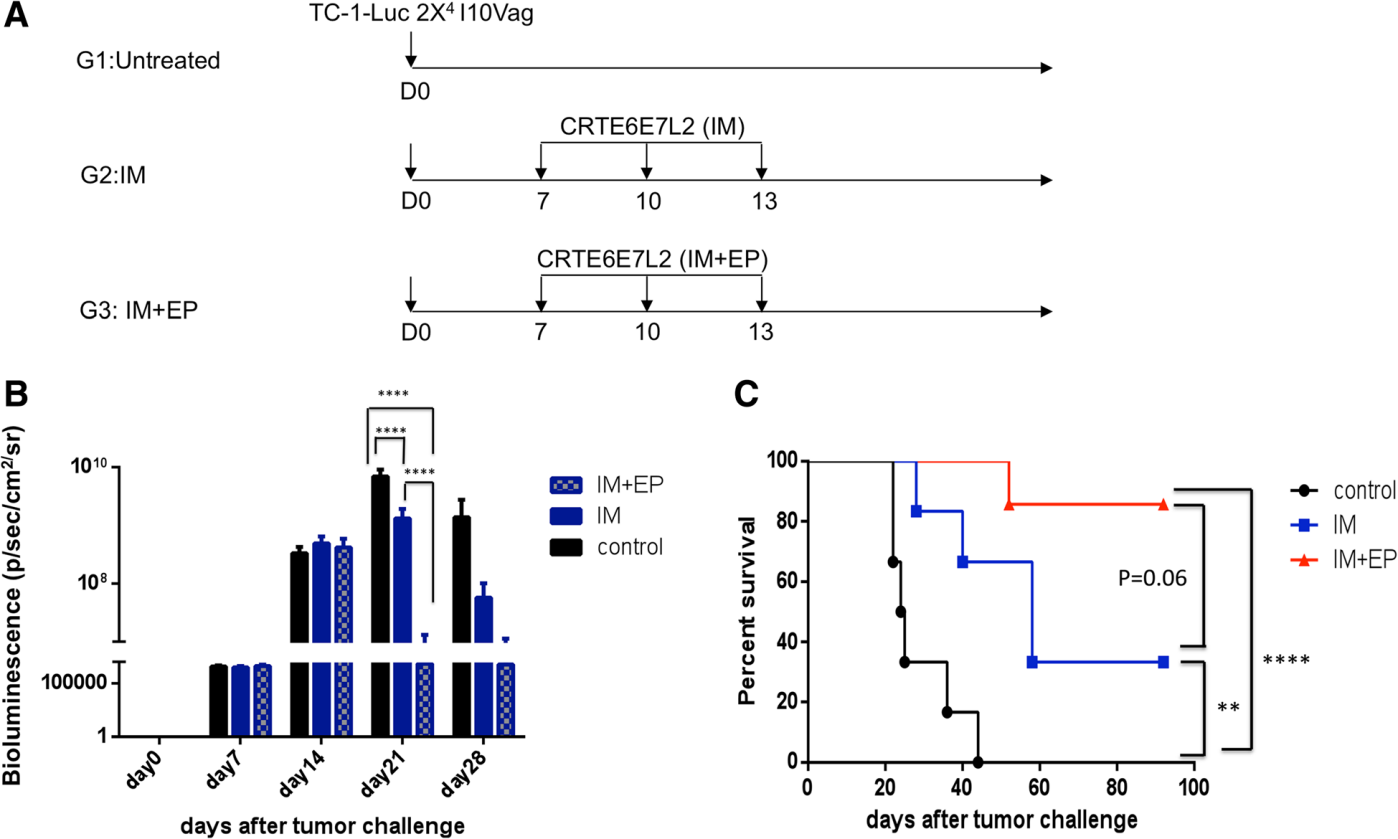
**Comparison of antitumor effect induced by CRT/E6E7L2 DNA vaccination with electroporation. (A)**. Schematic illustration of the experiment. C57BL/6 mice were (6-12 mice/group) were challenged intravaginally with 2x10^4^ TC-1 Luc cells. From day 7, mice were either left untreated or immunized with 10 μg/mouse of CRT/E6E7L2 DNA vaccine by intramuscular injection only or followed by electroporation. The mice were boosted twice with 3-day interval with the same regimen. **(B)** Tumor growth was followed every week by bioluminescence measurement (photons/sec/cm^2^). **(C)** Survival of the mice was monitored every other day.

C57BL/6 mice were challenged with firefly luciferase-expressing TC-1 tumor cells (TC-1-Luc) intravaginally. As shown in the treatment schedule in Figure 2A, mice were treated with CRTE6E7L2 DNA vaccine by IM administration with or without subsequent electroporation on days 7, 11 and 14 after tumor challenge. As shown in Figure 2B, IM administration of CRTE6E7L2 DNA vaccine followed by electroporation significantly reduced the intensity of luminescence indicating a reduction of tumor volume compared to IM vaccine without electroporation. Furthermore IM administration of CRTE6E7L2 DNA vaccine followed by electroporation prolonged survival compared to IM vaccine administration without electroporation (Figure 2C). These data indicate that electroporation significantly enhances the antitumor effects generated by the CRTE6E7L2 DNA vaccine.

### CRTE6E7L2 DNA vaccine administered intramuscularly followed by electroporation elicits antigen-specific CD8+ T cells systemically and locally

**Figure 3:**
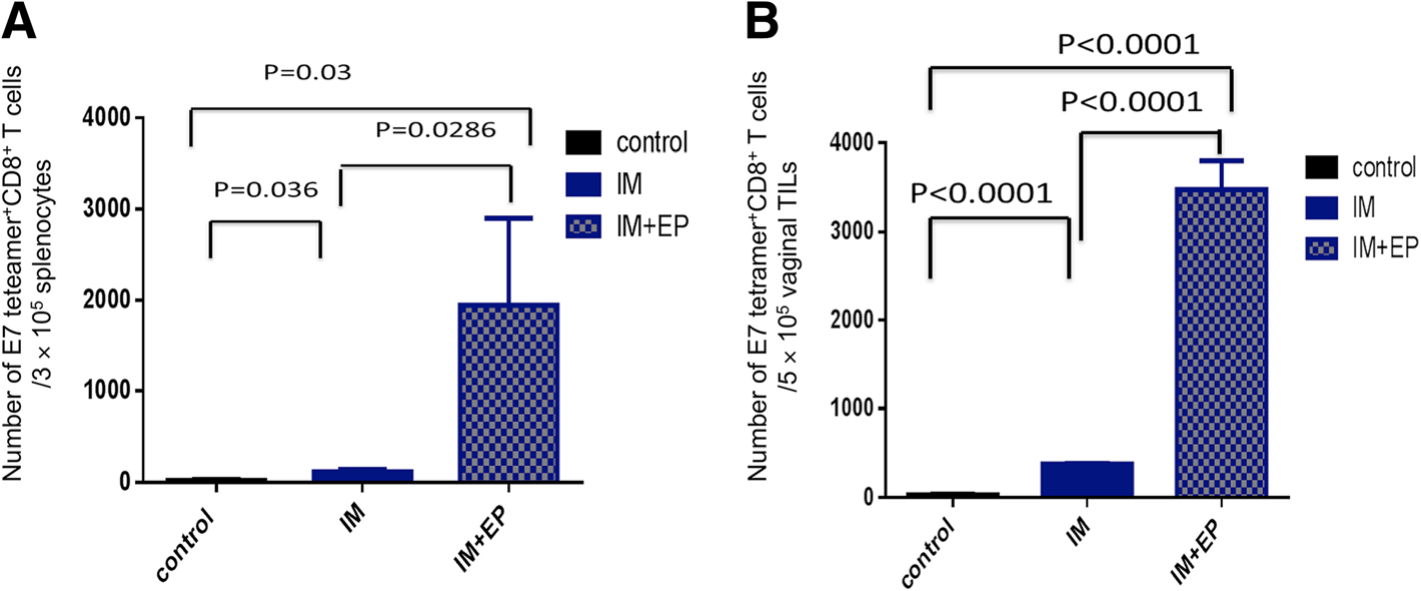
**Comparison of HPV16 E7-specific CD8**^**+**^**T cell responses induced by CRT/E6E7L2 DNA vaccination.** Summary of systemic **(A)** and tumor infiltrating **(B)** HPV16 E7-specific CD8^+^ T cells. Briefly, 5-8 week old female C57BL/6 mice were (5 mice/group) were challenged intravaginally with 2x10^4^ TC-1 Luc cells/mouse. From day 7, mice were either left untreated or immunized with 10 μg/mouse of CRT/E6E7L2 DNA vaccine by intramuscular injection only or followed by electroporation. The mice were boosted twice at 3-day intervals with the same regimen. 8 days after the last vaccination, splenocytes **(A)** or tumor infiltrating lymphocytes **(B)** were harvested and surface stained with anti-mouse CD8 and HPV16 E7aa49-57 peptide loaded H2-D^b^ tetramer. The cells were acquired with FACSCalibur.

Next, we examined which vaccine administration method most effectively generated E7-specific CD8+ T cells. Mice were challenged with TC-1-Luc tumor cells intravaginally and treated beginning one week later with three IM injections at three-day intervals of CRTE6E7L2 DNA vaccine either with or without subsequent electroporation. Splenocytes and tumor infiltrating lymphocytes were harvested 8 days after the last vaccination and analyzed for the presence of E7-specific CD8+ T cells. As shown in Figure 3A, IM injection of CRTE6E7L2 DNA vaccine followed by electroporation generated the highest percentage of E7-specific CD8+ T cells systemically. Furthermore, mice treated with CRTE6E7L2 DNA vaccine administered by IM injection followed by electroporation generated significantly greater E7-specific CD8+ T cells among vaginal tumor infiltrating lymphocytes compared to those treated with IM injection alone (Figure 3B). These data suggest that IM injection followed by electroporation is the better method of administration for the CRTE6E7L2 DNA vaccine compared to IM injection alone for the generation of antigen-specific CD8+ cell-mediated immune responses both systemically and locally.

### CRTE6E7L2 DNA vaccine generates potent protective antigen-specific immune responses and antitumor effects against E6/E7-expressing tumors in CD4-depleted mice

**Figure 4:**
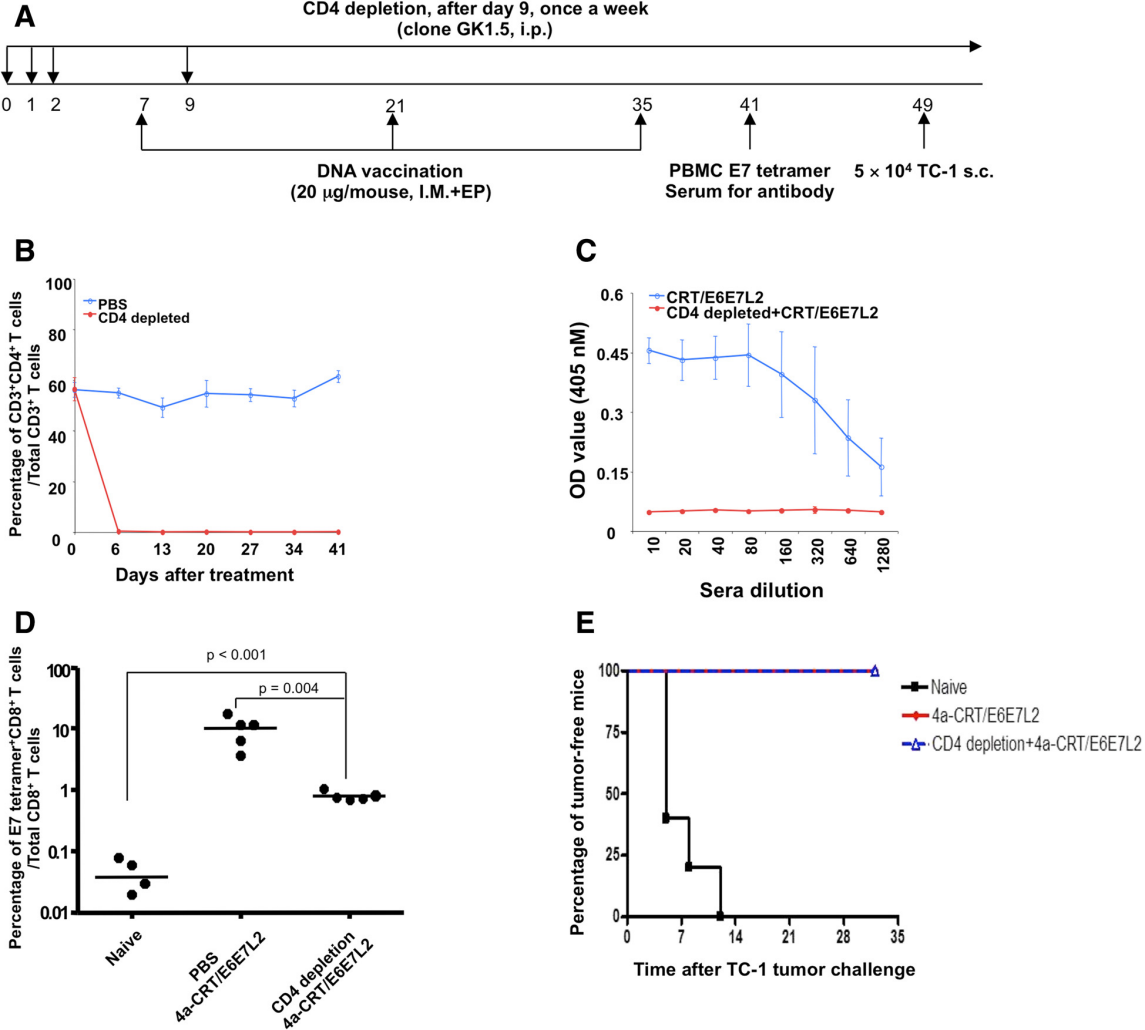
**Effect of CD4**^**+**^**T cell depletion on the immunogenicity of 4a-CRT/E6E7L2 DNA vaccine.(A)** Schematic diagram of the treatment schedule. Briefly, 5-8 weeks old female C57BL/6 mice (5 mice/group) treated with 200 μg/mouse of anti-mouse CD4 depletion antibody (clone GK1.5) or PBS via intraperitoneal injection for 3 days and the depletion was maintained by weekly injection of the depletion antibody (100 μg/mouse). **(B)** Success of CD4^+^ T cell depletion was verified by peripheral blood CD4 staining followed by flow cytometry analysis. 1 week after depletion initiation, mice were vaccinated with 20 μg/mouse of 4a-CRT/E6E7L2 DNA vaccine via intramuscular injection followed by electroporation. The mice were boosted with the same regimen twice at a 2-week interval. **(C)** One week after the last vaccination, serum was collected for the analysis of HPV16 E7-specific antibody response by ELISA. **(D)** HPV16 E7-specific CD8^+^ T cell responses were analyzed by staining PBMCs with HPV16 E7aa49-57 peptide loaded H2-D^b^ tetramer followed by flow cytometry analysis. **(E)** Two weeks after last vaccination, the mice were challenged with 5 × 10^4^ TC-1 cells subcutaneously. Tumor growth was monitored twice a week.

We found that vaccination of mice with CRT-E7(detox) DNA elicits a potent E7-specific CD8^+^ T cell response even in mice depleted for CD4+ T cells, suggesting that this vaccine may still be active even in CD4-depleted/HIV+ hosts (Additional file 1: Figure S1). Given our intent to also target E6 and L2, we further examined whether the CRTE6E7L2 DNA vaccine was effective in the generation of HPV antigen-specific T cell-mediated immune response as well as antitumor effects against E6/E7-expressing tumors under conditions of CD4+ T cell depletion. Mice were depleted of CD4+ T cells by administration of anti-mouse CD4 antibody on the schedule shown in Figure 4A, including prior to administration of CRTE6E7L2 DNA vaccine. The efficiency of CD4+ T cell depletion was verified by peripheral blood CD4 staining followed by flow cytometry analysis. As shown in Figure 4B, CD4+ T cells were completely depleted by day 6 after the initiation of antibody depletion. We first characterized the generation of HPV antigen-specific immune response by CRTE6E7L2 DNA vaccine with or without CD4 depletion using ELISA. We found that CD4 depleted mice treated with CRTE6E7L2 DNA vaccine had essentially no E7-specific antibody responses compared to non-depleted mice (Figure 4C), further suggesting successful ongoing CD4+ T cell depletion. In contrast, as shown in Figure 4D, CD4-depleted mice treated with CRTE6E7L2 DNA vaccine generated significantly greater E7-specific CD8 + T cells among total CD8+ T cells compared to naïve mice, although levels of E7-specific CD8+ T cells were lower than those in non-depleted mice. Nevertheless, the CRTE6E7L2 DNA vaccine conferred 100% protection against subcutaneous TC-1 tumor challenge in both CD4-depleted mice and non-depleted mice (Figure 4E). These data indicate that vaccination with CRTE6E7L2 DNA generated a potent HPV antigen-specific CD8+ T cell-mediated immune response, which translated into a protective antitumor effect in the absence of CD4+ T cells.

### CRTE6E7L2 DNA vaccine generates potent therapeutic antigen-specific immune responses and antitumor effects in CD4-depleted tumor-bearing mice

**Figure 5:**
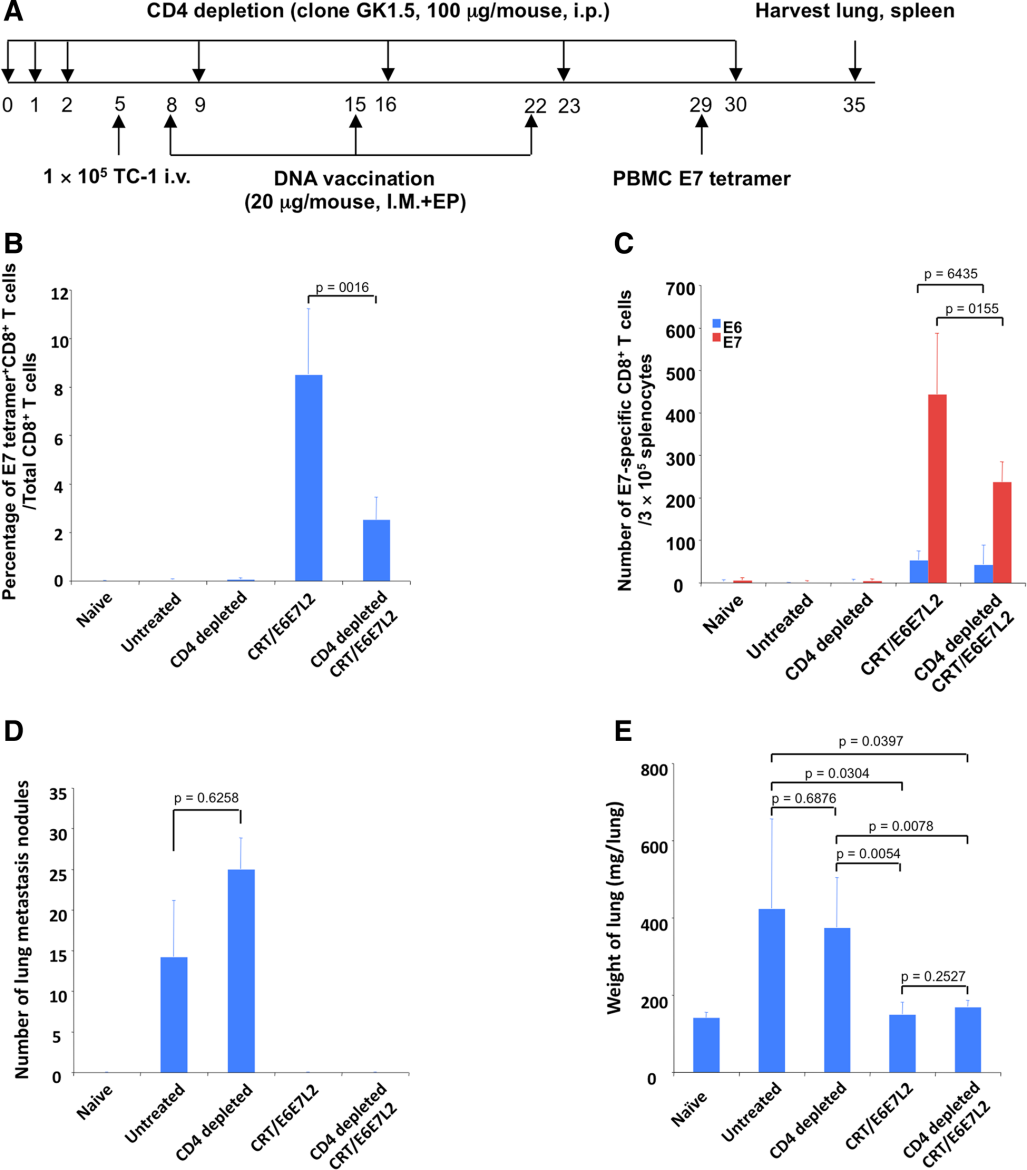
**Effect of CD4**^**+**^**T cell depletion on the therapeutic antitumor immunity generated by 4a-CRT/E6E7L2 DNA vaccine.(A)** Schematic illustration of the experiment. Briefly, 5-8 week old female C57BL/6 mice (5 mice/group) were treated with 100 μg/mouse of anti-mouse CD4 deletion antibody (clone GK1.5) or PBS via intraperitoneal injection for 3 days and the depletion was maintained by weekly injection of the depletion antibody (100 μg/mouse). 5 days after the initiation of the depletion, mice were injected with 1 × 10^5^ TC-1 cells intravenously. 3 days after TC-1 cell injection, mice were vaccinated with 20 μg/mouse of 4a-CRT/E6E7L2 DNA vaccine via intramuscular injection followed by electroporation. The mice were boosted with the same regimen twice with 1-week interval. **(B)** One week after last vaccination, PBMCs were collected and HPV16 E7-specific CD8^+^ T cell responses were analyzed by HPV16 E7aa49-57 peptide loaded H2-D^b^ tetramer staining followed by flow cytometry analysis. **(C)** 13 days after last vaccination, splenocytes were harvested for the detection of HPV-16 E6 and E7-specific CD8^+^ T cell responses analyzed by IFN-³ intracellular staining. Diagram of the number of TC-1 cell metastasis nodules **(D)** and the weight of lungs **(E)**.

Next, we tested whether the CRTE6E7L2 DNA vaccine could treat TC-1 tumor-bearing mice in CD4-depleted mice. Mice were depleted of CD4+ T cells in the priming phase and were challenged with TC-1 tumor cells by tail vein injection. Mice were then treated with CRTE6E7L2 DNA vaccine three times at one-week intervals by IM injection followed by electroporation (Figure 5A). In addition, DNA vaccinated mice without CD4+ T cell depletion were included for comparison. We found that CD4-depleted mice treated with CRTE6E7L2 DNA vaccine had a significantly greater percentage of E7-specific CD8+ T cells among total CD8+ T cells compared to naïve or untreated tumor-bearing mice (Figure 5B). Furthermore, the number of E6- and E7-specific CD8+ T cells among splenocytes was significantly greater in CD4-depleted mice treated with CRTE6E7L2 DNA vaccine compared to naïve or untreated tumor-bearing mice (Figure 5C). Importantly, CD4-depleted mice treated with CRTE6E7L2 DNA vaccine had zero lung metastasis nodules, as did non-depleted mice (Figure 5D). Additionally, CD4-depleted mice treated with CRTE6E7L2 DNA vaccine had significantly lower lung weight compared to untreated tumor-bearing mice with or without CD4 depletion (Figure 5E). Furthermore, DNA vaccinated mice with or without CD4 depletion demonstrated statistically similar lung weights (Figure 5E). These data suggest that the CRTE6E7L2 DNA vaccine is effective in treating TC-1 tumor-bearing mice despite the absence of CD4+ T cells.

## Discussion

In the current study, we examined the protective and therapeutic effects of CRTE6E7L2 DNA vaccine against E6/E7-expressing tumors in immunocompetent and immunosuppressed mice. We first demonstrated that administration of the CRTE6E7L2 DNA vaccine by IM injection followed by electroporation generated greater HPV neutralizing antibody titers and cell-mediated immune responses compared to IM injection alone. Although the use of alum enhanced the HPV neutralizing antibody titers, it was no more effective than electroporation alone, and the combination did not yield a clear advantage. In addition, the use of alum failed to enhance the HPV-specific cellular immune responses elicited by IM vaccination. IM injection of CRTE6E7L2 DNA vaccine followed by electroporation elicited more potent E7-specific CD8+ T cell responses and therapeutic antitumor effects in tumor-bearing mice compared to IM injection without electroporation, and these responses effectively targeted tumors in the vaginal tract. Notably, we also observed these potent cell-mediated immune responses and antitumor effects in CD4-depleted tumor-bearing mice. Taken together, our data suggest that CRTE6E7L2 DNA vaccine may be appropriate for use in immunocompromised patients, including HIV + patients, to control of HPV-16-associated cancer.

Here we observed that mice vaccinated with CRTE6E7L2 DNA vaccine were capable of generating potent HPV antigen-specific CD8+ T cell-mediated immune responses in the absence of CD4+ T cells. We have previously shown that DNA vaccines encoding CRT linked to the target antigen are capable of generating high levels of antigen-specific CD8^+^ T cell responses as well as significant antitumor immunity [14,15][21]. CRT is a heat shock protein that associates with peptides delivered into the ER [22] as well as with MHC class I-²2 microglobulin molecules to aid in antigen presentation [23]. Furthermore, CRT has been shown to bind with CD91 (also known as ±_2_-macroglobulin receptor or low density lipoprotein-related protein), a cell surface receptor on antigen presenting cells [24]. Thus, CRT may be able to deliver the linked antigens to dendritic cells through their specific binding with CD91 to facilitate cross priming activities. Furthermore, CRT has been shown to activate dendritic cells (DCs) [25].

A critical safety feature of the CRTE6E7L2 DNA vaccine is that it contains mutated (detox) versions of the E6 and E7 oncogenes. This is essential for the clinical translation of the CRTE6E7L2 DNA vaccine. Previously, it has been shown that mutation of E7 at position 24 and/or 26 disrupts the Rb binding site of E7 thus alleviating the concerns for the oncogenicity of E7 [26] and that mutation at position 91 destroys the single zinc finger in E7 [26]. This construct is being used in ongoing clinical studies. Additionally, mutation of E6 in positions that have been shown to destroy several key oncogenic functions, including the ability to bind p53, which prevent the E6 (detox) protein from immortalizing human epithelial cells [27,28], were included. We further deleted the PDZ protein-binding domain at the C-terminus of E6 (aa146-151) [29] as this is also critical for transformation. The DNA construct used in the current study includes HPV-16 E6 and E7 genes that contain the mutations described above, thus minimizing safety concerns for clinical translation.

In the clinical setting, DNA vaccines can be delivered by a variety of methods, which include particle-mediated intradermal delivery by gene gun, intralesional injection, intramuscular injection, and intramuscular injection followed by electroporation [30-33]. The gene gun enables delivered DNA to directly transfect keratinocytes and epidermal Langerhans cells (immature DCs). This stimulates DC maturation and migration to the local lymphoid tissue, where DCs prime T cells for HPV antigenâ€“specific immune responses. DNA vaccine administration with electroporation increases the number of HPV DNAâ€“transfected cells and enhances the magnitude of gene expression, while requiring less time to reach a maximal immune response compared to conventional intramuscular vaccine injection. The use of an electroporation device may be even more important in non-human primates and humans than mice [34]. Recent reports from clinical trials indicated that delivery of naked DNA vaccines with electroporation was capable of generating potent cellular as well as humoral immune responses against encoded HPV antigens [35]. Additionally, the CRT/E7 (detox) DNA vaccine, administered by IM injection and electroporation, is being tested in HPV-16-associated head and neck cancer patients (NCT01493154), as well as via intracervical injection or a gene gun like device (PMED) (NCT00988559).

The current study suggests the potential of CRTE6E7L2 DNA vaccination to treat HPV-16 infections or HPV-16-associated diseases in both HIV- and in HIV + patients. HIV + patients in particular need a preventive and therapeutic HPV vaccine because they are more susceptible to HPV infection and often have frequent multi-type infections including uncommon HPV subtypes. Furthermore, HIV + patients exhibit more severe and progressive HPV infections compared to healthy individuals. The CRTE6E7L2 DNA vaccine has the potential to fulfill this urgent need because it safe, effective in the absence of CD4+ T cells and elicits potent cell-mediated immune responses and therapeutic antitumor effects in murine studies.

## Methods

### Mice

5 ~ 8 week old female C57BL/6 mice were purchased from the National Cancer Institute (Frederick, MD). All mice were housed at Johns Hopkins University School of Medicine cancer center animal facility under specific-pathogen free conditions, and all procedures were performed according to approved protocols and in accordance with recommendations for the proper use and care of laboratory animals.

### Peptides, antibodies and regents

The H-2D^b^-restricted HPV16 E7aa49-57 peptide, RAHYNIVTF and H-2K^b^-restricted HPV16 E6aa50-57 peptide, YDFAFRDL, were synthesized by Macromolecular Resources (Denver, CO) at a purity of ≥ 80%. FITC-conjugated anti-mouse CD3 (clone 145-2C11), FITC, PE and APC-conjugated anti-mouse CD8a (clone 53.6.7), and FITC-conjugated anti-mouse IFN-³ (clone XMG1.2) antibodies were purchased from BD Pharmingen (BD Pharmingen, San Diego, CA). PE-conjugated anti-mouse CD4 (clone RM4-4) antibody was purchased from Biolegend (San Diego, CA). PE-conjugated, HPV16 E7aa49-57 peptide and RAHYNIVTF loaded H2-D^b^ tetramers were obtained from the National Institute of Allergy and Infectious Diseases Tetramer Facility (Atlanta, GA). Medroxyprogesterone acetate was purchased from Greenstone LLC (Peapack, NJ), and 4% Nonoxynol-9 (N-9) was purchased from Revive personal products company (Madison, NJ).

### Cells

HPV-16 E6 and E7-expressing TC-1 cells were generated as previously described [36]. TC-1-Luc cells were established by transducing TC-1 cells with luciferase. The cells were maintained in RPMI medium supplemented with 2 mM glutamine, 1 mM sodium pyruvate, 100IUml^−1^ penicillin, 100μgml^−1^ streptomycin and 10% fetal bovine serum (FBS). The creation of 293TTF and Lovo-T has been described [37] and cultured in DMEM medium containing 2 mM glutamine, 1 mM sodium pyruvate, 100IUml^−1^ penicillin, 100μgml^−1^ streptomycin and 10% FBS.

### DNA vaccine

The DNA vaccine, pNGVL4a-CRTE6E7L2 used in this study was constructed as follows. Briefly, CRTdE6E7L2 contains human calreticulin (CRT), E7 with three mutations [38], E6 with two mutations [38] and deletion of aa146-151 [29], and 11-200aa of HPV16 L2. To clone pNGVL4a-CRTdE6dE7L2, human CRT was isolated from pNGVL4a-hCRTE6E7L2 [21] by Sal I/EcoRI. DE6E7L2 codon, modified and synthesized by Genescript (Piscataway, NJ), was cut EcoRI/BamHI. The digested human CRT and dE6E7L2 were cloned into pNGVL4a vector digested with Sal/BamHI. The mutation and deletion sequences of E6 and E7 are detailed in Additional file 2: Figure S2.

### Electroporation-mediated DNA vaccination

pNGVL4a-CRT/E6E7L2 DNA vaccine was administered to C57BL/6 mice via intramuscular (IM) injection **in the flank** with or without subsequent electroporation with Ichor TriGridâ„¢ Electroporation Delivery System (TDS) (Ichor Medical Systems Inc., San Diego, CA) as described previously [9]. When aluminum phosphate was used, DNA was mixed with 22.5 μg of aluminum phosphate with a final volume of 50 μl. The DNA/aluminum phosphate mixture was used within half hour after preparation. When vaccination schedules required a booster vaccination, the contralateral leg was used for vaccination, and subsequent vaccinations used alternating hind legs.

### *In vivo* CD4^+^ T cell depletion

To deplete CD4^+^ T cells, mice were injected with a depleting anti-mouse CD4 antibody (Clone GK1.5) for 3 days via intraperitoneal injection. The depletion was maintained through the experiment by injecting the antibody once per week. The success of depletion was confirmed by staining peripheral blood cells with an anti-mouse CD4 antibody that recognizes a different epitope than the depleting antibody.

### Production of furin-cleaved HPV16 pseudovirus

Production of furin-cleaved HPV16 pseudoviruses (fcPsV) is described elsewhere [37]. Briefly, fcPsV were produced by following the standard PsV production protocol [39,40] with the following modifications: (1) instead of 293TT cells, 293TTF cells were used; (2) ammonium sulfate was excluded from the maturation buffer; (3) the concentration of CaCl_2_ in the maturation buffer was increased to 5 mM; and (4) maturation time was increased from 24 hours to 48 hours.

### *In vitro* HPV pseudovirus neutralization assay

The details of the *in vitro* HPV pseudovirus neutralization assay are described elsewhere [37]. Briefly, 1.5 × 10^4^ LoVoT cells were plated into 96-well tissue culture plate. The next day, mouse serum was serially diluted and mixed with fcPsV containing luciferase, incubated at 37°C for 2 hours before adding to the cells. These plates were then incubated at 37°C for 72 hours. Luciferase activity was analyzed and a 50% reduction in luciferase activity was considered as positive for neutralization.

### ELISA

HPV16 E7-specific antibody response was determined by an enzyme-linked immunosorbent assay (ELISA) as described previously [41] and the optical density (OD) value was read with xMark microplate spectrophotometer (BioRad, Hercules, CA) ELISA reader at 450 nm.

### Preparation of single-cell suspensions from spleens and TC-1/luciferase tumors

Single splenocyte suspensions were prepared by meshing spleens and lysing red blood cells. To prepare single cells from vaginal TC-1-Luc tumors, tumors were surgically resected under sterile conditions and placed in RPMI 1640 medium containing 100Uml^−1^ penicillin, 100μgml^−1^ streptomycin on ice, and washed with phosphate-buffered saline (PBS). The solid tumors were then minced into 1- to 2-mm pieces and incubated with serum-free RPMI 1640 medium containing 1mgml^−1^ collagenase D, and 0.25mgml^−1^ DNase I (both from Roche, Indianapolis, IN), 100Uml^−1^ penicillin, 100μgml^−1^ streptomycin and incubated at 37°C with periodic agitation. The cells were then filtered through a 70-μm nylon filter mesh to remove undigested tissue fragments. The resultant single tumor cell suspensions were washed twice in Hank’s buffered salt solution (HBSS) (400 *g* for 10 min), and viable cells were determined using Trypan blue dye exclusion.

### Tetramer staining

For tetramer staining, 1 × 10^6^ splenocytes or tumor infiltrating lymphocytes were stained with purified anti-mouse CD16/32 (Fc block, BD Pharmingen, San Diego, CA) first, and then stained with anti-mouse CD8-FITC, PE-conjugated, HPV16 E7aa49-57 peptide, RAHYNIVTF loaded H2-D^b^ tetramer at 4°C. After the wash, cells were stained with 7-AAD prior to flow cytometry analysis to exclude dead cells. The cells were acquired with FACSCalibur and analyzed with CellQuest (BD Bioscience, Mountain View, CA) or FlowJo software (Tree Star, Ashland, OR).

### Intracellular cytokine staining and flow cytometry analysis

To detect HPV16 E6 or E7-specific CD8^+^ T cell responses by IFN-³ intracellular staining, splenocytes were stimulated with either HPV16 E6aa50-57 or E7aa49-57 peptide (1 μg/ml) at the presence of GolgiPlug (BD Pharmingen, San Diego, CA) at 37°C overnight. The stimulated splenocytes were then washed once with FACScan buffer and stained with PE-conjugated monoclonal rat anti-mouse CD8a. Cells were subjected to intracellular cytokine staining using the Cytofix/Cytoperm kit according to the manufacturer’s instruction (BD Pharmingen, San Diego, CA). Intracellular IFN-³ was stained with FITC-conjugated rat anti-mouse IFN-³. Flow cytometry analysis was performed using FACSCalibur with CELLQuest software.

### *In vivo* tumor protection experiment

Mice were depleted of CD4^+^ T cells, mice were injected with a depleting anti-mouse CD4 antibody (Clone GK1.5) for 3 days via intraperitoneal injection as described above. Mice were then vaccinated with 20 μg pNGVL4a-CRT/E6E7L2 DNA by IM injection followed by electroporation three times at two-week intervals. 8 days after last vaccination, mice were injected with 5x10^4^ TC-1 cells subcutaneously. The tumor growth was monitored by palpation twice a week.

### *In vivo* tumor treatment experiment in a vaginal tumor model

To test whether pNGVL4a-CRT/E6E7L2 DNA vaccine-induced T cell responses could cure existing HPV16 E6E7-expressing tumors, an orthotopic vaginal tumor model was used. CD4 depletion was performed as described above. Briefly, female C57BL/6 mice were treated with 3 mg/mouse of medroxyprogesterone acetate via subcutaneous injection. 4 days later, the mice were treated intravaginally with 4% N-9 overnight. After wash, 2x10^4^ TC-1/luciferase cells were instilled into intravaginal cavity. Seven days after tumor challenge, mice were vaccinated with 10 μg pNGVL4a-CRT/E6E7L2 DNA by IM injection followed by electroporation three times at three-day intervals. Tumor growth was monitored by luminescence imaging at indicated time points.

### *In vivo* tumor treatment experiment in a hematologic spread model

After CD4^+^ T cell depletion, mice were injected with 1x10^5^ TC-1 cells intravenously via the tail vein. 3 days later, mice were vaccinated with pNGVL4a-CRT/E6E7L2 DNA vaccine via IM administration followed by electroporation and boosted twice with the same regimen at one-week intervals. 30 days after TC-1 cell challenge, mice were sacrificed, splenocytes were prepared to detect HPV-16 E6- and E7-specific CD8+ T cell responses and lungs were harvested to examine the tumor growth.

### Statistical analysis

All data were expressed as means ± standard deviations (SD). Comparisons between individual data point was analyzed by 2-tailed Student’s *t* test. Data for tumor treatment experiments were evaluated by analysis of variance (ANOVA). Survival distributions for mice in different groups were compared by the Kaplanâ€“Meier curves and by use of the long-rank tests. A *P* value of less than 0.05 was considered significant.

## Abbreviations

HPV: Human papillomavirus; HIV: Human immunodeficiency virus; CRT: Calreticulin; IM: Intramuscular; Luc: Luciferase; DC: Dendritic cell.

## Competing interests

Richard Roden is an inventor on L2 patents licensed to Shantha Biotechnics Ltd., GlaxoSmithKline, PaxVax, Inc. and Acambis, Inc. and has received research funding from Sanofi Pasteur and GlaxoSmithKline. Richard Roden and T.-C. Wu hold equity in Papivax LLC and serve as scientific advisors of Papivax Biotech Inc. The terms of these arrangements are managed by Johns Hopkins University in accordance with its conflict of interest policies. Yung-Nien Chang is an employee of Papivax LLC. Drew Hannaman is an employee and minority shareholder in Ichor Medical Systems, Inc.

## Authors’ contributions

SP, RA, RBSR, YNC, CFH and TCW conceived and designed experiments and interpreted data. SP, LS and JWW performed experiments. JK, RBSR, CFH and TCW wrote the manuscript. All authors read and approved the final manuscript.

## Supplementary Material

Additional file 1: Figure S1 HPV-16 E7 peptide loaded MHC class I tetramer staining to characterize the frequency of HPV-16 E7-specific CD8 + T cells in CRT/E7 DNA vaccinated mice with or without CD4 depletion. C57BL/6 mice (5 per group) were vaccinated with pcDNA-3 CRT/E7 DNA [14] intradermally via gene gun twice at 1-week interval using methods similar to what we have described previously [11]. Mice vaccinated with empty pcDNA-3 vector were included as controls. One group of CRT/E7 DNA vaccinated mice were depleted of CD4+ T cells 3 days before DNA vaccination and were continuously depleted of CD4+ T cells twice a week with mouse monoclonal antibody GK1.5 using methods described previously [11]. The completeness of the CD4+ T cell depletion was confirmed by flow cytometry analysis. 5 days after the last DNA vaccination, spleens from the vaccinated mice were harvested and characterized for the presence of HPV-16 E7-specific CD8+ T cells using HPV-16 E7 peptide (aa 49-57) loaded H-2 D^b^ tetramer staining [42] and CD8 staining followed by flow cytometry analysis. Click here for file

Additional file 2: Figure S2 Sequences of HPV-16 E6 and E7 (detox) antigens in pNGVL4a-hCRTE6E7L2 DNA vaccine. The sequences of the relevant sections of wild type (top) and detox (bottom) HPV-16 E6 and E7 are shown. Letters in red indicate mutations and dashes indicate deletions. Click here for file
