# Supplementary figures and images for: Control of HPV-associated tumors by innovative therapeutic HPV DNA vaccine in the absence of CD4+ T cells
Source: Cell Biosci. 2014 Mar 4;4:11. doi: 10.1186/2045-3701-4-11 (PMC4015858; doi:10.1186/2045-3701-4-11)

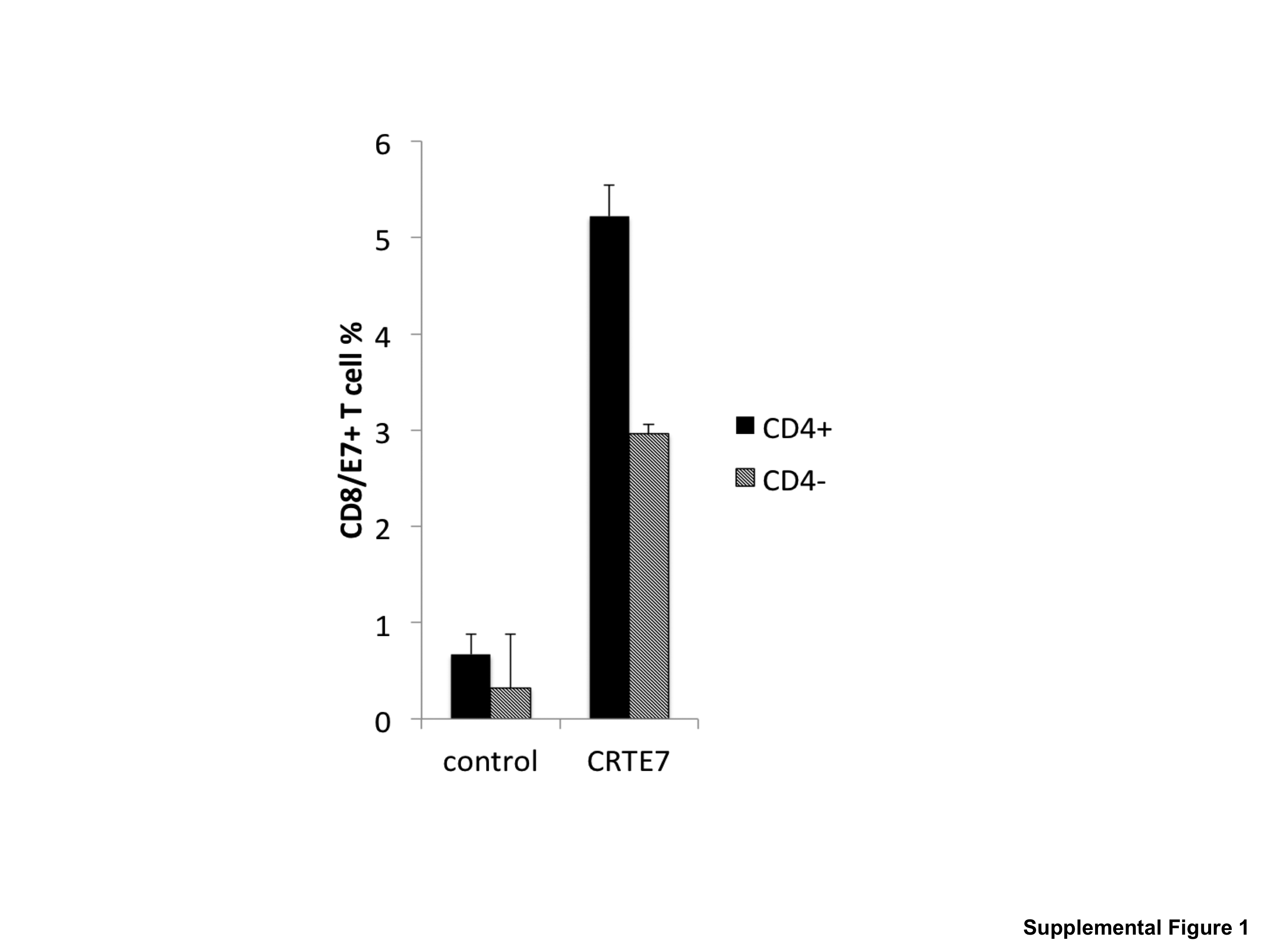

Supplement: Additional file 1: Figure S1 — HPV-16 E7 peptide loaded MHC class I tetramer staining to characterize the frequency of HPV-16 E7-specific CD8 + T cells in CRT/E7 DNA vaccinated mice with or without CD4 depletion. C57BL/6 mice (5 per group) were vaccinated with pcDNA-3 CRT/E7 DNA [14] intradermally via gene gun twice at 1-week interval using methods similar to what we have described previously [11]. Mice vaccinated with empty pcDNA-3 vector were included as controls. One group of CRT/E7 DNA vaccinated mice were depleted of CD4+ T cells 3 days before DNA vaccination and were continuously depleted of CD4+ T cells twice a week with mouse monoclonal antibody GK1.5 using methods described previously [11]. The completeness of the CD4+ T cell depletion was confirmed by flow cytometry analysis. 5 days after the last DNA vaccination, spleens from the vaccinated mice were harvested and characterized for the presence of HPV-16 E7-specific CD8+ T cells using HPV-16 E7 peptide (aa 49-57) loaded H-2 Db tetramer staining [42] and CD8 staining followed by flow cytometry analysis. [file 2045-3701-4-11-S1.tiff]

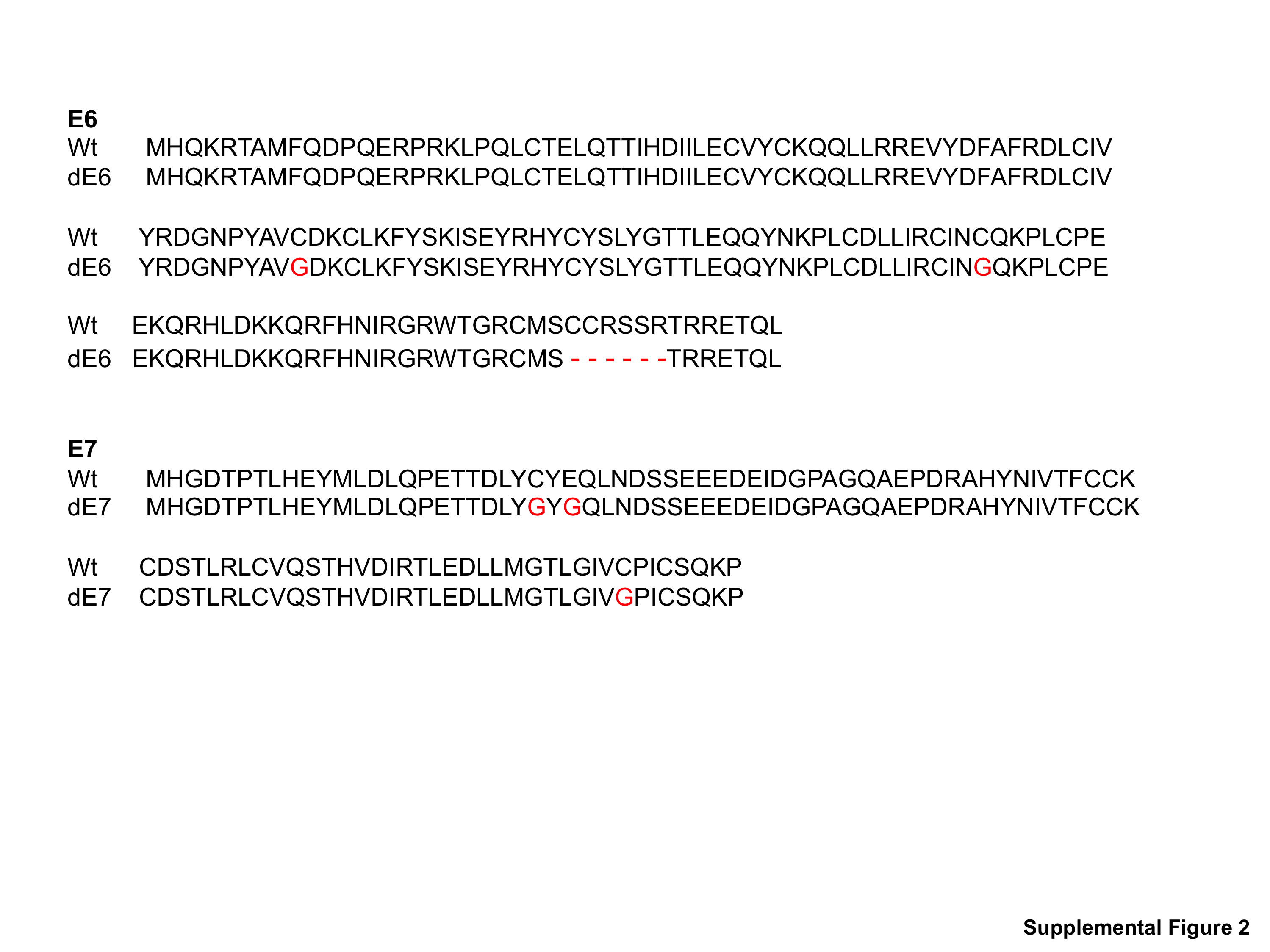

Supplement: Additional file 2: Figure S2 — Sequences of HPV-16 E6 and E7 (detox) antigens in pNGVL4a-hCRTE6E7L2 DNA vaccine. The sequences of the relevant sections of wild type (top) and detox (bottom) HPV-16 E6 and E7 are shown. Letters in red indicate mutations and dashes indicate deletions. [file 2045-3701-4-11-S2.tiff]
